# Clinical characteristics and management of hemodialysis patients with pre-dialysis hypertension: a multicenter observational study

**DOI:** 10.1080/0886022X.2022.2136527

**Published:** 2022-10-25

**Authors:** Yaoxian Liang, Liangying Gan, Yulan Shen, Weihua Li, Dongliang Zhang, Zhongxin Li, Jianwei Ren, Mingcheng Xu, Xiaolin Zhao, Yingchun Ma, Li Zuo, Mei Wang

**Affiliations:** a Department of Nephrology, Peking University People’s Hospital, Beijing, China; b Department of Nephrology, Miyun Hospital, Beijing, China; c Department of Nephrology, Shijingshan Hospital, Beijing, China; d Department of Nephrology, Peking University International Hospital, Beijing, China; e Department of Nephrology, Capital Medical University Affiliated Luhe Hospital, Beijing, China; f Department of Nephrology, Aviation General Hospital, Beijing, China; g Department of Nephrology, Zhanlanlu Hospital of Xicheng District, Beijing, China; h Jiaozhou Bethune Blood Purification Center, Shandong, China; i Department of Nephrology, Beijing Boai Hospital, China Rehabilitation Research Center, Beijing, China

**Keywords:** Hypertension, hemodialysis, pre-dialysis blood pressure, home blood pressure

## Abstract

**Background:**

Hypertension is a leading preventable risk factor for cardiovascular disease in hemodialysis patients. Pre-dialysis systolic blood pressure (SBP) more than 160 mmHg was thought to be associated with increased risk of cardiovascular events and all-cause mortality. The present study was performed to explore the clinical characteristics and management of hemodialysis patients with pre-dialysis SBP ≥ 160 mmHg.

**Methods:**

A total of 1233 patients undergoing hemodialysis from nine hemodialysis centers were enrolled. Pre-dialysis and home BP were measured and clinical data were collected. The characteristics of patients with pre-dialysis SBP ≥ 160 mmHg were explored. Clinical parameters between hypertensive and non-hypertensive patients were compared. The partial correlation analyses performed to identify the associations between BP and clinical parameters.

**Results:**

There were 24.6% of the hemodialysis patients had pre-dialysis SBP ≥ 160 mmHg and the average SBP was 173.8 ± 10.9 mmHg. Only 21.4% of the patients achieved dry weight after dialysis and up to 30.2% of patients were not given combination therapies of antihypertensive drugs. Compared to patients with pre-hemodialysis SBP < 160 mmHg, patients with pre-dialysis SBP ≥ 160 mmHg had lower target-reaching rate of Kt/v and higher incidences of intradialytic hypotension and muscle spasm. Most patients (96%) with pre-dialysis SBP ≥ 160 mmHg had home SBP≥ 135 mmHg. Patients with home SBP ≥ 160 mmHg had higher left ventricular weight index and lower hemoglobin levels when compared to their counterparts with home SBP <160 mmHg.

**Conclusions:**

Pre-dialysis SBP ≥ 160 mmHg is common in clinical practice and most of the patients could diagnosed to be hypertensive according to their home SBP. Patients with pre-dialysis SBP ≥ 160 mmHg are more likely to be subjected to dialysis insufficiency and intradialytic complications. Achieving dry weight and sufficient pharmacologic interventions should be strengthened to improve BP control in the hemodialysis population.

## Introduction

The number of patients with end stage renal disease (ESRD) requiring hemodialysis is rapidly increasing in recent years [1]. The high hospitalization and mortality rates of hemodialysis patients place a heavy burden on the national healthcare system in China [2,3]. Hypertension is a well-established modifiable risk factor for cardiovascular disease, which is the leading cause of death among patients with ESRD undergoing chronic hemodialysis [4]. Although current guidelines call for blood pressure (BP) control as a top priority in dialysis patients, hypertension is prevalent in a vast majority of patients on dialysis and often remains poorly controlled. Compared to general population, BP management in dialysis patients is more challenging because of the complexity of BP measurement and the lack of global standard criteria for defining hypertension [5,6].

Blood pressure readings that are obtained pre-dialysis are commonly used to make therapeutic decisions by clinicians. According to the Kidney Disease Outcomes Quality Initiative clinical practice guideline, hypertension in hemodialysis patients is diagnosed when pre-dialysis BP is > 140/90 mmHg or when post-dialysis BP is > 130/80 mmHg [7]. However, the use of conventional pre-dialytic BP recordings to diagnose hypertension has been a matter of debate. Emerging data have indicated that pre-dialytic BP has unclear prognostic value for cardiovascular events and all-cause mortality in dialysis patients [8,9]. As a matter of fact, published data on this area are conflicting. Several recent studied suggested that pre-dialysis BP was associated with risk factors of cardiovascular disease and mortality among patients on hemodialysis [10–13]. Currently, pre-dialysis BP is still widely used as a practical way to manage hypertension by nephrologists in many hemodialysis units. Previous works on this subject mainly focused on the association between pre-dialysis BP and mortality and morbidity in hemodialysis patients, as well as the optimal pre-dialysis BP at which the outcomes risk was at its lowest [8–14]. However, the clinical characteristic of patients with pre-dialysis hypertension and factors associated with the development of pre-dialysis hypertension have not been fully investigated. Also, effective management of pre-dialysis hypertension is warranted.

According to the Dialysis Outcomes and Practice Patterns Study (DOPPS), patients with pre-dialysis systolic BP (SBP) ≥ 160 mmHg is considered to be associated with higher risk of cardiovascular events and all-cause mortality [14]. Therefore, patients with pre-dialysis SBP ≥ 160 mmHg always deserves more attention in clinical practice. In the present study, we focus on the clinical characteristics of hemodialysis patients with pre-dialysis SBP ≥ 160 mmHg and probe therapeutic strategies of hypertension. In addition, we also evaluated home BP recordings of the subjects and their association with clinical parameters.

## Methods

### Study population

This is an observational study including maintenance hemodialysis patients from 9 hemodialysis centers in China. Adult patients (>18 years) were eligible to participate to the study if they: (i) had ESRD treated with hemodialysis for >3 months; (ii) were following a standard thrice-weekly hemodialysis schedule; and (iii) provided an informed written consent. Exclusion criteria included: (i) arteriovenous fistula in bilateral brachial arms that could interfere with arm BP measurement; (ii) modification of dry weight or anti-hypertensive treatment during 2 weeks prior to enrollment; and (iii) severe cardiovascular diseases (myocardial infarction, angina, arrhythmia, heart failure) or stroke during 4 weeks before enrollment. This study was conducted ethically in accordance with the Declaration of Helsinki and its revisions. The study protocol was approved by the Ethics Committee of Peking University People’s Hospital. Written informed consents were obtained from all participants in this study.

### Blood pressure measurement and data acquisition

All subjects underwent pre-hemodialysis BP measurement over two weeks with the use of a validated oscillometric device at the level of brachial artery in the non-fistula arm, after 5 min of rest and with two measurements per occasion taken 2 min apart, according to guidelines. Patients were asked to record their home BP when the two-week average pre-hemodialysis SBP ≥ 160 mmHg. Home BP was obtained using a validated self-inflating automatic oscillometric device (Omron Healthcare). Patients themselves or their family members were instructed in the use of this monitor. For each patient, home BP was recorded three times daily (on waking up, between noon and 6 p.m., and at bedtime) over six non-dialysis days and were averaged. A series of demographic parameters, clinical data, laboratory tests, dialysis-related parameters and complications were recorded.

### Statistical analysis

Statistical analysis was performed with Statistical Package for Social Sciences 22.0. Continuous variables are presented as mean ± standard deviation or median with interquartile range according to the normality of distribution, and categorical variables as frequencies and percentages (*n*, %). Normality of distribution for quantitative variables was examined using the Kolmogorov–Smirnov or the Shapiro–Wilk test. For continuous variables, student’s *t*-test or one-way ANOVA was used when the data were normally distributed, while the Mann–Whitney *U*-test was used when the data were not normally distributed. The χ2-test was applied for dual comparisons of categorical variables. The partial correlation analyses were performed to identify the associations between BP and clinical parameters and to adjust for confounding variables. Values of *p* < .05 (two-tailed) were considered statistically significant in all comparisons.

## Results

### Baseline characteristics

**Figure 1.**
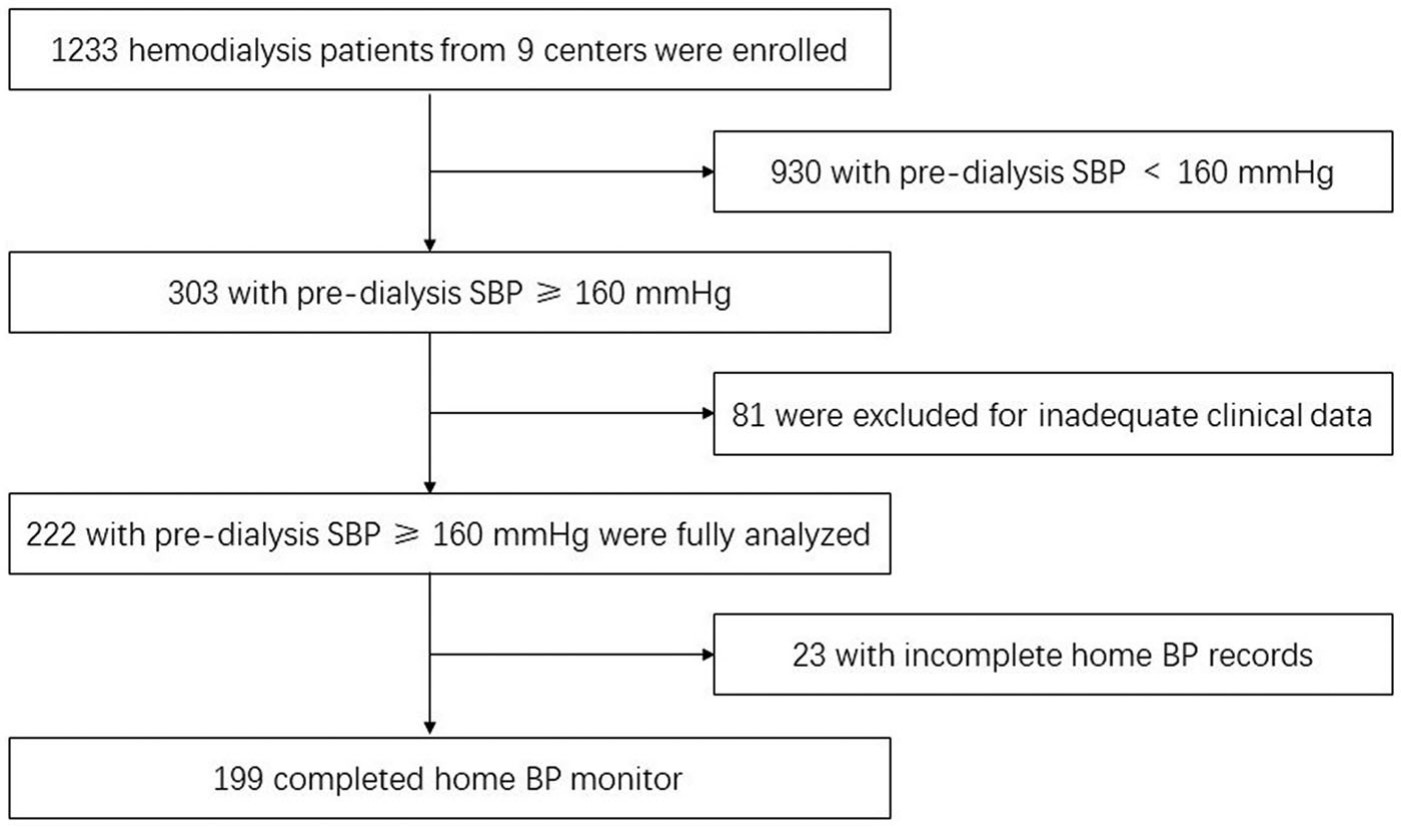
Flow diagram of the study.

A total of 1233 maintained hemodialysis patients from nine hemodialysis centers were enrolled in the study, 303 (24.6%) of which with pre-dialysis SBP ≥ 160 mmHg. Of these, 81 patients had inadequate clinical data, and 222 patients (137 men and 85 women) were fully analyzed. The flow diagram of the study was shown in Figure 1. The average pre-hemodialysis SBP was 173.8 ± 10.9 mmHg. The mean age of these participants was 58.6 ± 13.5 years and the median hemodialysis vintage was 44.0 (21.5–73.0) months. Diabetic nephropathy was the most common cause of ESRD, followed by glomerulonephritis. The median inter-dialytic weight gain (IDWG) was 3.13 kg (4.7% of dry weight) and 21.6% patients achieved dry weight after dialysis. The baseline demographic, clinical, laboratory and hemodialysis-related parameters of the participants are presented in Table 1.

**Table 1.** Characteristics of patients with pre-dialysis SBP ≥ 160 mmHg (*n* = 222).

Variables	Value
Male/Female (*n*)	137/85
Age (years)	58.6 ± 13.5
Diabetes (*n*, %)	124, 55.9%
Hemodialysis vintage (month)	44 (21.5–73.0)
Pre-dialysis SBP (mmHg)	173.8 ± 10.9
Pre-dialysis DBP (mmHg)	84.7 ± 11.9
Post-dialysis SBP (mmHg)	153.1 ± 20.2
Post-dialysis DBP (mmHg)	82.2 ± 13.1
Home SBP (mmHg)	162.1 ± 13.6
Home DBP (mmHg)	82.2 ± 11.0
UF with one dialysis session (Kg)	2.72 ± 0.86
UF/dry weight (%)	4.1 ± 1.2
IDWG (Kg)	3.13 (2.47–3.83)
IDWG/dry weight (%)	4.7 ± 1.1
Achievement of dry weight (%)	21.6%
Hypotension during dialysis (%)	12.2%
Muscle spasm during dialysis (%)	6.3%
Edema (%)	17.6%
Serous effusion (%)	6.8%
LVWI	214.30 ± 109.96
LVDd (mm)	49.7 ± 5.4
LVEF (%)	64.5 ± 8.3
BNP (pg/ml)	438.00 (260.00–814.00)
Kt/v	1.38 ± 0.28
Pre-dialysis SCr (umol/L)	886.2 ± 242.0
Pre-dialysis sodium (mmol/L)	137.1 ± 4.3
Pre-dialysis potassium (mmol/L)	5.09 ± 0.84
Pre-dialysis CO2CP (mmol/L)	22.27 ± 2.42
Hemoglobin (g/L)	110.8 ± 12.2
Albumin (g/L)	39.57 ± 3.28
Pre-dialysis Calcium (mmol/L)	2.21 ± 0.22
Pre-dialysis Phosphorus (mmol/L)	1.75 ± 0.53
iPTH (pg/ml)	214.00 (114.30–361.19)
Type of antihypertensive drugs (*n*, %)	
CCBs	185, 83.3%
β-blockers	108, 48.6%
ACEIs/ARBs	101, 45.5%
α-blockers	55, 24.8%
Loop diuretics	10, 4.5%
Number of antihypertensive drugs	
Median (*n*)	2 (1–3)
0 (*n*, %)	10, 4.5%
1 (*n*, %)	57, 25.7%
2 (*n*, %)	80, 36.0%
3 (*n*, %)	50, 22.5%
4 (*n*, %)	19, 8.6%
5 (*n*, %)	6, 2.7%

ACEIs: angiotensin converting enzyme inhibitors; ARBs: angiotensin II receptor blockers; BNP: B-type natriuretic peptide; CCBs: calcium channel blockers; CO2CP: carbon dioxide combining power; DBP: diastolic blood pressure; IDWG: inter-dialytic weight gain; iPTH: intact parathyroid hormone; LVDd: left ventricular end-diastolic dimension; LVEF: left ventricular ejection fraction; LVWI: left ventricular weight index; SBP: systolic blood pressure; SCr: serum creatinine; UF: ultrafiltration.

Comparisons between patients with pre-dialysis SBP ≥ 160 mmHg and <160 mmHg. Compared to patients with pre-hemodialysis SBP < 160 mmHg, the percentage of patients achieving a Kt/V not less than 1.2 was lower in patients with pre-hemodialysis SBP ≥ 160 mmHg. No significant differences were observed in the target-reaching rate of hemoglobin, albumin, serum calcium, serum phosphorus and intact parathyroid hormone (iPTH) in these two groups (Table 2). Moreover, patients with pre-dialysis SBP ≥ 160 mmHg are more likely to suffer from acute complications related to dialysis such as hypotension and muscle spasm (Table 2).

**Table 2.** Comparisons of the target-reaching rate of clinical parameters and the incidence of acute complications in patients with pre-dialysis SBP ≥ 160 mmHg and < 160 mmHg.

	Pre-dialysis SBP ≥ 160 mmHg	Pre-dialysis SBP < 160 mmHg	χ^2^	*p* value
Kt/v	78.6%	87.9%	11.765	.001
Hemoglobin	54.8%	52.3%	0.464	.496
Albumin	45.0%	47.9%	0.613	.434
Calcium	68.9%	74.7%	3.062	.08
Phosphorus	55.7%	52.5%	0.740	.390
iPTH	30.4%	30.6%	0.004	.951
Hypotension during dialysis	12.2%	6.8%	10.362	.000
Muscle spasm during dialysis	6.3%	1.6%	27.421	.000

Recommended targets: Kt/v, no less than 1.2; hemoglobin, 110–130 g/L; albumin, no less than 40 g/L; calcium, 2.10–2.54 mmol/L; phosphorus, 0.87–1.45 mmol/L; iPTH, 150–300pg/ml.

### Home BP in this population

There were 199 patients with pre-dialysis SBP ≥ 160 mmHg completed home BP monitor and the average home SBP was 162.1 ± 13.6 mmHg, which was significantly lower than pre-dialysis SBP (*p* < .001). There were 124 (62.3%) subjects had home SBP ≥ 160 mmHg and 191 (96.0%) had home SBP≥ 135 mmHg. After adjusted for age, hemodialysis vintage, and serum sodium concentration, Home SBP was positively correlated with pre-dialysis SBP, post-dialysis SBP, left ventricular weight index (LVWI), B-type natriuretic peptide (BNP), and negatively correlated with hemoglobin. While pre-dialysis SBP had poor correlation with clinical parameters (Table 3).

**Table 3.** Partial correlations between SBP and clinical parameters.

	Pre-dialysis SBP		Home SBP	
	Partial correlation coefficient	*p* value	Partial correlation coefficient	*p* value
Pre-dialysis SBP	–	–	0.328	.000
Post-dialysis SBP	0.206	.007	0.283	.000
Home SBP	0.328	.000	–	–
LVWI	0.216	.242	0.597	.000
BNP	−0.052	.559	0.181	.042
Hemoglobin	−0.051	.510	−0.193	.012

Adjusted by age, hemodialysis vintage, and serum sodium concentration.

We further compared clinical data between patients with home SBP ≥ 160 mmHg and < 160 mmHg. As shown in Table 4, patients with home SBP ≥ 160 mmHg had higher pre- and post-dialysis BP, higher LVWI, BNP, and lower hemoglobin levels.

**Table 4.** Comparisons of clinical parameters in patients with home SBP ≥ 160 mmHg and < 160 mmHg.

	Home SBP ≥ 160 mmHg (*n* = 124)	Home SBP < 160 mmHg (*n* = 75)	*p* value
Pre-dialysis SBP (mmHg)	174.7 ± 11.5	169.6 ± 8.8	.001
Post-dialysis SBP (mmHg)	156.5 ± 19.7	146.4 ± 18.6	.000
Kt/v	1.38 ± 0.28	1.40 ± 0.26	.512
Hemoglobin (g/L)	108.7 ± 12.6	113.5 ± 10.7	.008
Albumin (g/L)	39.64 ± 3.08	39.66 ± 2.91	.969
Pre-dialysis Sodium (mmol/L)	136.7 ± 4.8	137.5 ± 3.3	.239
Pre-dialysis Calcium (mmol/L)	2.22 ± 0.21	2.22 ± 0.18	.798
Pre-dialysis Phosphorus (mmol/L)	1.79 ± 0.51	1.65 ± 0.58	.077
iPTH (pg/ml)	195.90 (114.40–363.67)	225.20 (100.00–417.00)	.929
LVEF (%)	64.0 ± 9.2	65.1 ± 6.3	.553
LVDd (mm)	50.5 ± 5.7	48.0 ± 4.3	.053
LVWI	249.34 ± 102.88	160.39 ± 101.42	.021
BNP (pg/ml)	504.50 (306.97–885)	313.00 (224.50–549.8)	.012
Number of antihypertensive drugs	2.3 ± 1.1	2.1 ± 1.1	.060

### Antihypertensive medications

**Figure 2.**
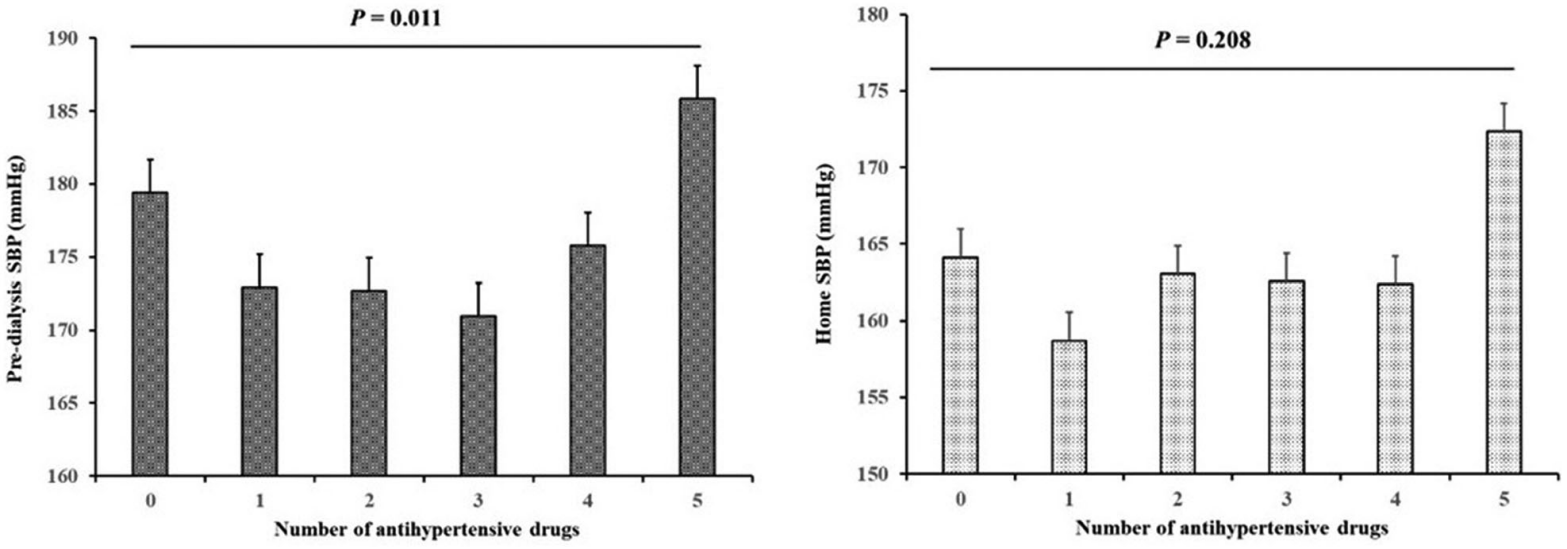
Pre-dialysis SBP and home SBP in patients with different numbers of antihypertensive drugs. Patients receiving five antihypertensive drugs had the highest levels of both pre-dialysis SBP and home SBP, followed by patients without antihypertensive agent.

Antihypertensive drugs were prescribed in 212 (95.5%) patients and 155 (69.8%) received two or more classes, while there were 10 (4.5%) patients with no antihypertensive drugs. Calcium channel blockers (CCBs) were the most commonly used antihypertensive drugs in these patients, followed by β-blockers (Table 1). Patients receiving five antihypertensive drugs had the highest pre-dialysis SBP and home SBP. Both pre-dialysis SBP and home SBP were higher in patients without antihypertensive agent when compared to patients taking 1–4 antihypertensive drugs (Figure 2).

## Discussion

In the present study, we explored the clinical characteristics of patients with pre-hemodialysis SBP ≥ 160 mmHg. The prevalence of pre-dialysis SBP ≥ 160 mmHg was 24.6% in hemodialysis patients, among whom there were quite a few patients failed to achieve dry-weight or didn’t receive adequate pharmacological treatment. Also, the target-reaching rate of Kt/v was lower while the incidence of intradialysis hypotension and muscle spasm was higher in these patients. Most patients with pre-dialysis SBP ≥ 160 mmHg had home SBP≥ 135 mmHg. Increased home SBP was associated with higher LVWI and lower hemoglobin, whereas pre-dialysis SBP levels were not.

In previous studies, a U-shaped association was observed between pre-dialysis SBP and clinical outcomes among patients on dialysis [10,11,15]. Data from the DOPPS showed that the optimal pre-dialysis SBP range was 130 to 160 mmHg [14]. Patients with pre-dialysis SBP more than 160 mmHg have an increased risk of cardiovascular events and all-cause mortality [14]. Although pre-dialysis BP is not recommended by international guidelines for the diagnosis and management of hypertension in patients on hemodialysis, it is easy to obtain and now still used by vast majority of nephrologists in clinical practice. In this study, we found that 24.6% of subjects had pre-dialysis SBP ≥ 160 mmHg, indicating the poor hypertension control in hemodialysis patients. Furthermore, it should be noted that an overwhelming majority of patients with pre-dialysis SBP over 160 mmHg in this study had home SBP≥ 135 mmHg, and more than 60% of whom had home SBP≥ 160 mmHg. Also, correlation analysis revealed a significant positive correlation between home SBP and pre-dialysis SBP. These data indicate that there is a favorable consistency between pre-dialysis BP and home BP, highlighting the importance of pre-dialysis BP measurements. Clinical physicians should pay more attention to those with pre-dialysis SBP over 160 mmHg and strengthen the monitoring of home BP or 44-h ambulatory BP.

Volume overload is considered to be a prominent pathogenic mechanism of hypertension in patients with chronic hemodialysis [16,17]. Increasing percentage of inter-dialytic weight gain is associated with increases in pre-dialysis BP [18,19]. In the observational study, we found that the median IDWG was up to 4.7% of dry weight in patients with pre-dialysis SBP ≥ 160 mmHg. Even worse, only a small number of patients could achieve dry weight after the dialysis session. Furthermore, the target-reaching rate of Kt/v was lower in these patients when compared to their counterparts with pre-dialysis SBP < 160 mmHg. These results indicate that fluid overload and dialysis inadequacy might be partly responsible for the poor control of hypertension in patients with pre-dialysis SBP ≥ 160 mmHg. Consistent with our findings, previous studies have demonstrated that higher pre-dialysis BP is associated with increasing percentage of IDWG and lower dialysis Kt/v in subjects undergoing conventional hemodialysis [19,20]. Restriction of IDWG is critical to achieve dry weight and reduce acute complications related to dialysis such as hypotension and muscle spasm, thus is beneficial for BP control.

Pharmacologic treatment is another important approach for the management of hypertension in patients on dialysis. Meta analyses suggest that BP lowering with the use of antihypertensive agents is associated with reduced cardiovascular morbidity and mortality in dialysis patients [21,22]. A combination therapy of different classes of antihypertensive drugs is often required in ESRD patient due to the difficulty of blood pressure control [23]. In our survey, however, up to 30.2% of participants with pre-dialysis SBP ≥ 160 mmHg were prescribed none or only one antihypertensive drug. Furthermore, both pre-dialysis SBP and home SBP were much higher in patients taking no antihypertensive medication. Considering the poor management of hypertension in these patents, we suggest that aggressive pharmacologic intervention is warranted.

Compared with BP recordings obtained pre-dialysis, home BP represents a more powerful predictor of clinical outcomes and exhibits stronger associations with mean 44-h ambulatory BP monitoring, which is considered to be gold standard method for diagnosing hypertension in patients on dialysis. Thus, home BP monitoring is strongly recommended by international guidelines for the diagnosis and management of hypertension in dialysis population [24,25]. According to the consensus by the European Renal and Cardiovascular Medicine working group, hypertension in hemodialysis patients is diagnosed when home BP is ≥ 135/85 mmHg [25]. In the present study, we confirmed that SBP measured pre-dialysis was significantly higher than the average home SBP. Previous studies have reported that pre-dialysis BP recordings are higher than 44-h or 48-h ambulatory BP by a variable amount [26,27]. Therefore, BP would be generally overestimated and the management might be problematic when using pre-dialysis BP to diagnose hypertension [26]. Several factors may lead to the inaccuracy of pre-dialysis BP, such as volume expansion, accumulation of uremic toxins, and white coat effect [28,29]. Additionally, patients with higher home SBP showed higher LVWI and BNP. Home SBP but not pre-dialysis SBP is associated with LVWI and BNP. These results are in agreement with previous studies that home BP monitoring provides superior prognostic information of further cardiovascular events compared to peridialytic BP recordings [25,30,31], highlighting the role of home SBP as a predictor of target organ damage and mortality in patients on chronic hemodialysis.

An interesting finding of our study was that home SBP was negatively correlated with hemoglobin levels. The exact relationship between BP and anemia in patients on dialysis has not been well established. However, hypertension is a common complication of erythropoietin treatment and higher doses of erythropoietin stimulating agents always lead to a higher BP response [32,33]. It is a limitation that data of erythropoietin therapy was not collected in this study. We interpret with caution that large doses of erythropoietin stimulating agents used in patients with lower hemoglobin might partly contribute to the poor control of home BP.

There are several other limitations to our report. First, it is a cross-sectional observational study, preventing us from clarifying the association between pre-dialysis hypertension and clinical outcomes such as cardiovascular events and mortality. Second, demographic and clinical data of patients with pre-dialysis SBP < 160mmHg were not fully collected, and therefore not all the characteristics were compared between patients with pre-dialysis SBP ≥160mmHg and SBP <160mmHg. However, we analyzed the differences of several important clinical parameters and intradialytic complications between the two groups. Finally, defined daily dose of antihypertensive drugs were not assessed in this study, which might be confounding factors that account for the pre-dialysis hypertension.

In summary, the present investigation demonstrates the characteristics of hemodialysis patients with pre-dialysis SBP more than 160 mmHg. Most of the subjects could diagnosed to be hypertensive according to their self-recorded BP at home. The important implications of pre-dialysis BP should not be overlooked and close attention is required to the patients with pre-dialysis SBP over 160 mmHg. Home BP exhibits stronger associations with the presence of target organ damage and therefore might be prognostically more informative than pre-dialysis BP. Minimizing volume overload by control of IDWG and sufficient pharmacotherapy should be strengthened in the management of hypertension among these hemodialysis patients.

## Data Availability

All data generated or analyzed during the current study are included in this article.
